# Identification of Hub Genes and Target miRNAs Crucial for Milk Production in Holstein Friesian Dairy Cattle

**DOI:** 10.3390/genes14112105

**Published:** 2023-11-20

**Authors:** Zahra Roudbari, Morteza Mokhtari, Abdolvahab Ebrahimpour Gorji, Tomasz Sadkowski, Ayeh Sadat Sadr, Masoud Shirali

**Affiliations:** 1 Department of Animal Science, Faculty of Agriculture, University of Jiroft, Jiroft 7867155311, Iran; roudbari.zahra@ujiroft.ac.ir (Z.R.); msmokhtari@ujiroft.ac.ir (M.M.); 2 Department of Physiological Sciences, Institute of Veterinary Medicine, Warsaw University of Life Science, 02-776 Warsaw, Poland; abdolvahab_ebrahimpourgorji@sggw.edu.pl (A.E.G.); tomasz_sadkowski@sggw.edu.pl (T.S.); 3 Preclinical Modelling of Paediatric Cancer Evolution, Molecular Pathology Division, The Institute of Cancer Research, London SW7 3RP, UK; ayeh.sadr@icr.ac.uk; 4 Agri-Food and Biosciences Institute, Belfast BT9 5PX, UK; 5 School of Biological Sciences, Queen’s University Belfast, Belfast BT9 5DL, UK

**Keywords:** milk production, gene ontology, miRNA identification, network analysis

## Abstract

Dairy milk production is a quantitative trait that is controlled by many biological and environmental factors. This study employs a network-driven systems approach and clustering algorithm to uncover deeper insights into its genetic associations. We analyzed the GSE33680 dataset from the GEO database to understand the biological importance of milk production through gene expression and modules. In this study, we employed CytoNCA and ClusterONE plugins within Cytoscape for network analysis. Moreover, miRWalk software was utilized to detect miRNAs, and DAVID was employed to identify gene ontology and pathways. The results revealed 140 up-regulated genes and 312 down-regulated genes. In addition, we have identified 91 influential genes and 47 miRNAs that are closely associated with milk production. Through our examination of the network connecting these genes, we have found significant involvement in important biological processes such as calcium ion transit across cell membranes, the BMP signaling pathway, and the regulation of MAPK cascade. The conclusive network analysis further reveals that *GAPDH*, *KDR*, *CSF1*, *PYGM*, *RET*, *PPP2CA*, *GUSB*, and *PRKCA* are closely linked to key pathways essential for governing milk production. Various mechanisms can control these genes, making them valuable for breeding programs aiming to enhance selection indexes.

## 1. Introduction

In the realm of human nutrition, cow’s milk and its products have always been revered as essential foods. Milk, often hailed as a complete food, is renowned for its rich array of nutrients vital for the human body [1]. Neglecting the consumption of dairy products can result in irreversible consequences. For instance, according to Giosuè et al., cardiovascular health is more influenced by specific food types like cheese, yogurt, and milk rather than moderate dairy consumption [2]. Additionally, research by Babio et al. reveals an inverse relationship between total dairy product consumption and obesity [3]. As a result, milk stands as a crucial component of a healthy diet throughout various stages of life [3].

Traits related to milk include milk production rate, milk protein percentage, milk fat percentage, casein, and lactose percentage. The production potential of these traits is mainly controlled by different genes and environmental factors [4]. In addition, changes in milk production and composition have attracted the attention of dairy cattle breeders; hence, it is important to study genes controlling milk production and composition to identify and develop marker-assisted selection and optimize breeding programs to improve production traits [5]. Interactions between genes, proteins, and other substances in a cell form a complex network that has an important role in cell function. By unraveling these relationships and interdependencies, we can gain a deeper comprehension of milk production with desirable properties, encompassing elements like fat, lactose, protein, vitamins, micronutrients, and more [6].

Using different bio database systems can help to access lots of genetic information and protein structure; so, by identifying genes related to milk production traits, we can design new approaches to increase milk production or by gene manipulations modulate its content, following consumers’ demands [5]. Development in molecular technologies such as microarray techniques, such as Next-Generation Sequencing, along with advanced bioinformatics and data analytics approaches, has made it possible to study the expression of thousands of genes simultaneously, and this technology has become one of the most powerful tools in studying gene behavior and model gene regulatory networks; gene-regulatory networks are a collection of genes that interact indirectly with each other and with other molecules in the cell and control gene expression levels [7]. Among the aforementioned molecules are miRNAs, short, single-stranded RNA fragments of about 22 nucleotides that can be found in all cells and body fluids with a major role in regulating genetic processes. They can affect different processes such as proliferation, differentiation, growth, cell development, and immune response by regulation of gene expression [8]. These molecules are also vital in the process of mammary gland development and influence the milk production process as well [9].

The primary objective of this study is to identify genes that are differentially expressed (DEGs) along with their corresponding miRNAs. Additionally, this study intends to ascertain gene networks and cluster genes that work together in biological pathways related to milk production. Such an approach will facilitate future research in this area and will be the basis for cattle breeders to orientate breeding toward more efficient dairy production.

## 2. Materials and Methods

### 2.1. Microarray Data Pre-Processing and Statistical Analysis

The data utilized for analysis in this research were extracted from the GEO database [10] under the designated accession number GSE33680 (https://www.ncbi.nlm.nih.gov/geo/query/acc.cgi?acc=GSE33680/; accessed on 9 October 2023). 

It is important to highlight that the Animal Rights principles were not applicable to this project, as it did not involve any direct interaction or use of animals. The experiment consisted of comparing the transcriptomes of mammary glands from four samples of healthy lactating Holstein Friesian cows with high milk yield (average 11,097 kg milk/lactation) and four samples of low milk yield (average 6956 kg milk/lactation). Gene expression was measured using the GPL11648 Agilent-023647 Bos taurus (Bovine) Oligo Microarray platform. Before gene expression analysis, raw data underwent quality control. Criteria for identifying genes with significant differential expression DEGs were identified using GEO2R were (|log2 fold-change (FC)| > 2) and adj *p*-value < 0.05. GEO2R, accessible at http://www.ncbi.nlm.nih.gov/geo/geo2r/ (accessed on 9 October 2023), is a web-based tool designed to facilitate the comparison of two or more groups of samples within a GEO dataset, helping researchers pinpoint genes that exhibit differential expression under varying experimental conditions. GEO2R accomplishes this by examining the raw microarray data provided by the original submitters, employing the GEOquery and limma R packages from the Bioconductor project. In the current investigation, we utilized GEO2R to identify Differentially Expressed Genes (DEGs). The gene set enrichment analysis, gene ontology terms (GO), and Kyoto Encyclopedia of Genes and Genomes pathways (KEGG) were identified using the Database for Annotation, Visualization and Integrated Discovery (DAVID, https://david.ncifcrf.gov/, accessed on 9 October 2023) [11]. 

### 2.2. Network Module Creation Using the Integration of Protein–Protein Interactions (PPIs)

After identifying DEGs (up and down-regulated genes) and genes associated with milk production, the STRING database (https://string-db.org/, accessed on 9 October 2023) was utilized to retrieve interacting genes/proteins. This database is a reliable tool for obtaining protein interaction network information [12]. For the visualization network, Cytoscape software was employed, where nodes represent proteins and edges represent their interactions.

To identify key genes in the network, three methods were employed: degree centrality (DC), betweenness centrality (BC), and closeness centrality (CC). These topology scores were calculated using the CytoNCA plugin [13]. Nodes with high-grade centrality scores were identified as key genes in the milk production interaction network.

For further analysis, the Cluster ONE plugin in Cytoscape [14] was utilized to explore sub-network modules within the PPI network. This approach allowed us to identify functionally related gene clusters, shedding light on potential pathways and mechanisms involved in milk production. By integrating these methods and tools, we gained valuable insights into the complex interactions between genes and proteins relevant to milk production, paving the way for a more profound comprehension of the regulatory mechanisms governing this essential biological process.

### 2.3. miRNAs, Biological Processes, and Pathway Analysis of DEGs

One of the most common and reliable approaches to investigating complex interaction regions is through miRNA identification [15]. To explore the dense regions of the network, we employed the miRWalk tool to identify the target miRNAs for each gene. Specifically, some miRNAs that have an important role in milk production, as proven in other studies, were selected. Finally, for further analysis, we conducted biological process (BP) and pathway analysis using the DAVID database.

### 2.4. Relevance Network Design

To explore the connections between genes, miRNAs, and various biological processes, including milk production and lactation, we utilized Pathway Studio Web (Elsevier, Amsterdam, The Netherlands; https://www.pathwaystudio.com/; accessed on 9 October 2023). This platform allowed us to design an intergene interaction network. We visualized the relationships between all differentially expressed genes using Pathway Studio’s Build Pathway functionality, which relies on the wave propagation algorithm developed for navigating complex networks.

## 3. Results

### 3.1. DEGs Identification and PPI Analysis

**Figure 1:**
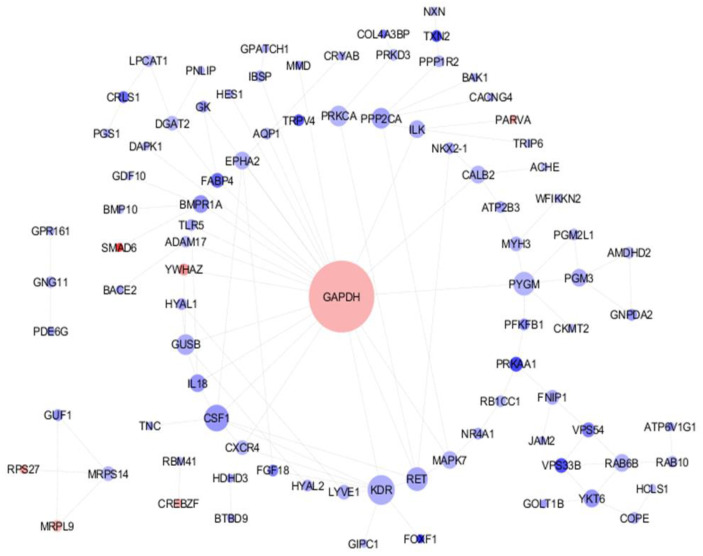
Protein–protein Interaction (PPI) network of genes that exhibit differential expression and are closely associated with milk production. The size of each node is drawn in proportion to its degree value, with greater dimensions indicating higher degrees. Furthermore, the color of each node is indicative of gene expression, with up-regulated genes shaded blue and downregulated genes shaded red.

In the initial phase of analysis, the raw data quality was carefully assessed. The investigation of differential gene expression was conducted using GEO2R. The present study has effectively determined a comprehensive set of 452 genes that exhibit statistically significant differences in their expression levels between the mammary glands of high- and low-milk-production Holstein Friesian cows. Out of the total 452 genes examined, it is noteworthy to mention that 140 genes exhibited up-regulation while the remaining 312 genes showed down-regulation. To gain insights into the functionality of the DEGs, the DAVID functional enrichment analysis highlighted 91 genes that were particularly relevant to milk production. Subsequently, these genes were inputted into the STRING database to produce network associations among these candidate genes. This comprehensive approach may provide valuable clues regarding the molecular mechanisms underlying milk yield variations in Holstein Friesian cows and contribute to our understanding of the genetic factors influencing milk production traits. In this network, there are 89 nodes with an average node degree of 1.94, indicating that, on average, each node in the network is connected to approximately 1.94 other nodes. Notably, the interaction score between PARVA and ILK, as well as GNPDA2 and AMDHD2, was found to be 0.99, suggesting a strong or high level of interaction between the pairs of nodes mentioned. Moreover, the software utilized in this study helped identify an interaction network among genes. In the current network, the GAPDH gene plays a crucial role as it has been observed to be up-regulated. This observation suggests that GAPDH may have the potential to regulate other genes during the milk production process (Figure 1). 

### 3.2. Network Analysis

Investigation of dense areas of the interaction network using Cluster ONE in Cystoscope software revealed four significant sub-network modules in the milk production network in which 34 DEGs are engaged (*p*-value < 0.05) (Table 1). 

Furthermore, using the CytoNCA plugin and considering the median values for degree centrality, betweenness centrality, and closeness centrality, we successfully identified eight candidate targets. The detailed topological features of these targets are presented in Table 2. These candidate targets represent potentially crucial elements within the network.

### 3.3. Biological Processes, Pathway Analysis, and miRNA Identification

The use of the DAVID tool allowed the identification of biological processes activated by clustered genes. The analysis showed that clustering substantially enriches various biological processes, including translation, regulation of the MAPK cascade, and regulation of the cell cycle (Table 3). 

Furthermore, the pathway analysis of the clustered genes indicated their significant enrichment in pathways such as the ribosome, amino sugar and nucleotide sugar metabolism, and the Rap1 signaling pathway. The ribosome pathway is vital for the synthesis of proteins, which is a fundamental process in all living organisms. It has a significant impact on milk production because ribosomes in mammary gland cells are responsible for synthesizing milk proteins. The presence of genes enriched in this pathway indicates their contribution to the production of milk proteins. Amino sugar and nucleotide sugar metabolism pathways are involved in the biosynthesis and modification of sugars, which are important components of various cellular processes. These sugars serve as building blocks for glycoproteins, glycolipids, and proteoglycans, which are essential for milk production and lactation. The Rap1 signaling pathway is a complex network involved in various cellular processes, including cell growth, differentiation, and signal transduction. It has been implicated in the regulation of mammary gland development and lactation. The enrichment of genes in this pathway suggests their potential role in regulating milk production. These findings highlight the involvement of these genes in crucial cellular activities and pathways related to milk production and suggest their potential regulatory roles in these processes (Table 4).

**Figure 2:**
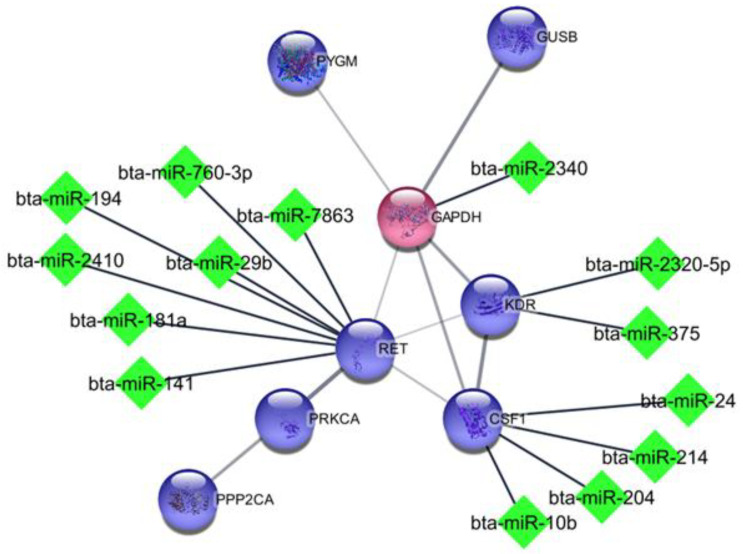
Interaction among hub genes and identified miRNAs. Green diamond shape is shown for miRNA; blue and red circles are shown for up- and down-regulated genes, respectively.

Using miRWalk software, we identified the miRNAs associated with hub genes. For further analysis, only miRNAs with established roles in milk production, supported by the existing literature, were chosen. Table 5 displays the 47 miRNAs that influence each of the hub genes and related genes from sub-network modules. The crucial genes for milk production, determined through statistical analysis, along with their respective miRNAs, were subjected to gene ontology analysis and are visually presented in Figure 2.

## 4. Discussion

Approximately 6875 genes are implicated in milk production in mammals. While certain genes are exclusively expressed in mammary glands, others are found to be active in multiple tissues, including the liver, kidneys, muscles, and more [16]. The study of genes involved in milk production can be an important step in identifying and developing marker-assisted selection and breeding programs to increase milk production in the dairy industry, which can also have a noteworthy impact on the economic landscape [5]. 

**Figure 3:**
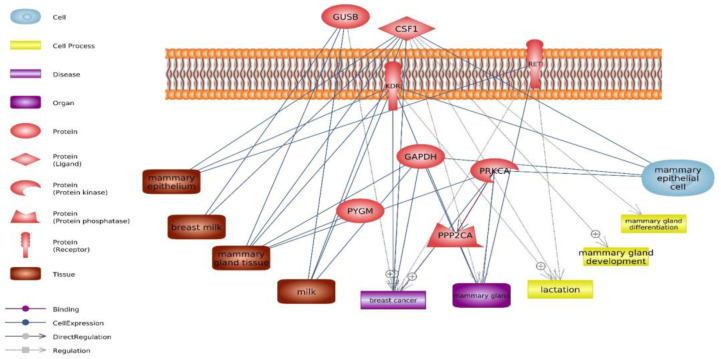
The interrelation among hub genes and their connection with factors associated with milk production, including tissue, organ, cell, and biological process.

Some factors, such as specific biological processes and pathways, along with genes and miRNAs, play a crucial role in determining the amount of milk produced. Understanding the genetic regulation and molecular mechanisms underlying milk synthesis can provide valuable insights for improving milk production in mammals, including dairy cattle. This study used various bioinformatics tools to identify hub genes involved in milk production. Through the application of Cluster ONE and CytoNCA (Table 1, Table 2, Table 3, Table 4 and Table 5) for network analysis, 8 hub genes were discovered, including *GAPDH*, *KDR*, *CSF1*, *PYGM*, *RET*, *PPP2CA*, *GUSB*, and *PRKCA* (Table 2, Figure 3). Genes with significantly different expressions can be identified as biomarkers—milk production traits. On the other hand, hub genes identified in this study, based on their interaction with other genes, can have different functions in biological processes, cellular components and molecular functions [17,18]. Different expressions in each of these key genes can significantly affect the function of other genes and proteins and influence multiple biological pathways such as SNARE interactions in vesicular transport, Rap1 signaling pathway, Ras signaling pathway, PI3K-Akt signaling pathway and MAPK signaling pathway. 

### 4.1. Hub Genes Related to Milk Production

According to network analysis, the *GAPDH* gene shows the highest degree in the network (Figure 1). This gene plays a key role in network stability and is an important gene in somatic cells. It is also one of the most common genes in mammary gland cells, and it has different expression in the different stages of lactation [19]. The expression of this gene in early lactation is 2 or 3 times higher than in late pregnancy. *GAPDH* is responsible for facilitating the oxidative phosphorylation of glyceraldehyde-3-phosphate to 1,3-bisphosphoglycerate during the process of glycolysis. Furthermore, it can reverse this reaction in glycogenesis-associated tissues. *GAPDH* is also involved in DNA replication and repair and apoptosis. It is expressed in subcutaneous adipose tissue and has a special role in fat metabolism in cattle, especially before and after calving when cows need to release fat stores for milk production [20]. 

Increased expression of the *KDR* gene during lactation leads to escalating metabolic activities. This gene has higher expression in the early lactation period [21]. The *CSF1* gene serves as a regulator of milk production and is prominently expressed in breast tissue during the stages of puberty, pregnancy, and lactation. Its involvement in the normal growth of mammary glands was proven (Sapi, 2004) [22]. *CSF1* is a tyrosine kinase receptor and secretes glycoproteins and proteoglycans. It is involved in phosphorylation activity and the PI3K biological pathway—which is important in the milk production process [23].

The obtained results are consistent with other studies and suggest that the *RET* gene is involved in cell growth and differentiation and the production of milk proteins [24]. *PPP2CA* gene is another important gene that encodes the catalytic subunit of alpha isoforms of serine phosphatase enzyme and threonine with 309 amino acids. Serine and threonine protein phosphatases represent a class of enzymes that facilitate the process of dephosphorylation on serine and threonine residues via hydrolysis of phosphoric acid monoesters [25]. It is also one of the most important phosphatases in insulin target cells. The expression of this gene influences plasma fatty acid concentration. In addition, the up-regulation of *PPP2CA* leads to an increase in the dephosphorylation of insulin receptor tyrosine and insulin receptor substrate, and thus downstream insulin messaging is reduced. A reduction in insulin signaling results in a decrease in the activation of PKB, a protein kinase with a crucial function in glucose uptake. Research has demonstrated the involvement of this gene in the increase in lipid and triglyceride levels in the blood [25]. Pawłowski et al. investigated mammary gland transcriptome and proteome in early lactation of Holstein cows and found involvement of *PPP2CA* in the following processes: protein catabolic process, regulation of inflammatory response, and RNA splicing [26].

Increased activation of the *GUSB* gene in this study corroborates earlier findings described in the available literature. This gene encodes beta-glucuronidases of the glycosidase family, which are enzymes that catalyze the breakdown of complex carbohydrates. This enzyme is also present in milk [27]. 

The findings of the current study are consistent with those of Paraboschi et al., who found that the *PRKCA* gene is required for calcium activation and has a significant role in many signaling pathways that are intricately engaged in the processes of cell proliferation, apoptosis, and cellular differentiation [28]. The expression of this gene has increased in epithelial cell growth in vitro and is involved in the expression of casein protein as well as the inhibition of mammary gland growth [29]. 

The *PYGM* gene is involved in the production of the enzyme phosphorylase. This enzyme regulates glycogenolysis and breaks down glycogen into glucose-1-phosphate, which eventually produces glucose–simple sugar, the major energy source for most cells. One of the pathways associated with this gene is PI3K-Akt, which is involved in galactose metabolism [30]. The PI3K-Akt pathway is considered one of the most vital pathways in biological systems, which affects alveolar cell proliferation, breast tissue development, milk production biosynthesis, amino acid metabolism, and the physiology and biology of milk production [31]. Furthermore, it can be expected that these genes have an impact on milk production and might be considered in the breeding program. 

### 4.2. miRNAs Related to Milk Production

It is important to identify miRNAs that can modify the expression of hub genes that have a crucial role in the control of milk production. Using the miRWalk software, we were able to find miRNAs with an impact on hub genes (Table 3). Among them, there are several described earlier in the literature as regulating the development of the mammary gland and milk production.

Some miRNAs identified in this research were also found by van Herwijnen et al. in their investigation on mammary gland cells, which revealed that bta-miR-141, miR-181a, bta-miR-375, bta-miR-22-3p are miRNAs found in the milk of cattle, humans, and pigs [32], what indicates their important role in milk production. This finding is in agreement with Luoreng et al., who studied mammary glands infected with *Staphylococcus aureus* and *Escherichia coli* and identified changed expression of some miRNAs such as bta-miR-181a, bta-miR-10b, bta-miR-214, bta-miR-375, bta-miR-146a, bta-miR-22-3p, bta-miR-2357, bta-miR-27a-5p, bta-miR-431, and other miRNA such as bta-miR-2344, bta-miR-7863, bta-miR-541, bta-miR-1247-3 and bta-miR-370 not identified in this research [33] (Figure 2). 

**Figure 4:**
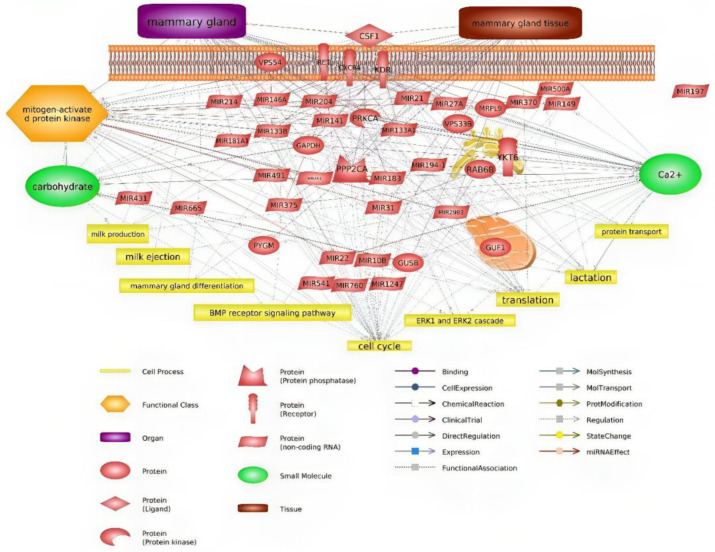
Relevance network of all identified DEGs, miRNAs, and biological processes.

Pawlowski et al. studied differences in miRNA expression between mammary epithelial cells and milk fat globules and showed that miR-141-3p and miR-204 were detected in the mammary tissue and mammary epithelial cells [26]. These miRNAs can regulate lactation via the expression of *STAT5* protein, which plays a crucial role in the mammary gland by influencing various pathways that fall under the influence of lactogenic and galactopoietic hormones [34]. It should be mentioned that in this investigation, we showed that miR-141-3p can regulate *RET*, *KDR*, *CXCR4*, and *PPP2CA* proteins (Figure 4).

The results obtained from the present study are congruent with the research conducted by Schanzenbach et al., who investigated the possibility of applying miRNAs such as bta-miR-194, bta-miR-2410, bta-miR-2340 and bta-miR-21-3p, that play a key role in milk production and can be used as biomarkers, for bovine early pregnancy diagnosis [35]. Moreover, The present findings seem to be consistent with other research conducted by Li et al., who found miRNAs in mammary glands of dairy cattle in response to heat stress (bta-miR-760-3p, bta-miR-2320-5p, bta-miR-2413, bta-miR-149-5p, bta-miR-491, bta-miR-183 and bta-miR-500) [36] indicating that these are miRNAs important from the point of view of the functioning of the mammary gland. 

It should also be mentioned that Menon et al. used the same accession number (GSE33680) in their investigation of milk production in buffalo breeds, namely: Banni, Jafrabadi, and Mehsani. In this study, authors used the MultiCom method to discern SNPs that are exclusive to buffaloes with high and low milk yields. Additionally, authors performed a comparative analysis with the cattle genome and demonstrated that certain pathways, including protein processing in the endoplasmic reticulum, MAPK signaling pathway, and ErbB signaling pathway, were identified as being the most noteworthy among the high-yield group [37], which also confirms in part the results obtained in our study.

## 5. Conclusions

Overall, considering the importance of the expression and function of genes affecting milk production, the objective of the present investigation was to discern some key genes in milk production using bioinformatic tools. Identified genes, such as *GAPDH*, *KDR*, *CSF1*, *PYGM*, *RET*, *PPP2CA*, *GUSB*, and *PRKCA*, are involved in milk production pathways and can be used in breeding programs. Despite the many complexities involved in understanding the mechanism of miRNA target gene selection, the use of specific patterns and algorithms allows identification and understanding of their function, as well as in milk production-related processes. The biological clustering of genes identified in this investigation genes may open a new perspective on those affecting milk production, their interdependencies, and regulation by miRNAs. A summary of the interactions between genes and miRNAs is shown in Figure 4, along with the biological processes in which they are involved.

## Figures and Tables

**Table 1:** The sub-network module obtained from Cluster ONE.

No.	Genes	*p*-Value
Cluster 1	*GUF1*, *MRPS14*, *MRPL9*, *RPS27*, *YKT6*, *RAB6B*, *VPS54*, *VPS33B*	5.8 × 10^−9^
Cluster 2	*GAPDH*, *KDR*, *CSF1*, *PYGM*, *GUSB*, *CXCR4*, *FGF18*, *FABP4*, *GDF10*, *BMP10*, *SMAD6*	3.4 × 10^−10^
Cluster 3	*PPP2CA*, *PRKCA*, *CACNG4*, *MAPK7*, *CCNI*, *BAK1*, *ANKLE2*, *PRKCA*	3.26 × 10^−7^
Cluster 4	*PRKAA1*, *AMDHD2*, *GNPDA2*, *PGM3*, *PGM2L1*, *PFKFB1*, *PDE6G*	0.000151

**Table 2:** Hub genes and ranks of degrees based on CytoNCA.

Gene	Gene Name	Expression Log FC	Degree	Betweenness	Closeness
*GAPDH*	glyceraldehyde-3-phosphate dehydrogenase	−1.07139	22	4384.83	0.074
*KDR*	Kinase insert domain receptor	−0.99096	8	539.66	0.070
*CSF1*	Colony-stimulating factor1	−0.49033	8	180.5	0.070
*PYGM*	phosphorylase, glycogen, muscle associated	−0.4587	6	2199.0	0.072
*RET*	Ret proto-oncogene	−0.39795	6	397.33	0.071
*PPP2CA*	Protein phosphatase 2 catalytic subunit alpha	0.66955	5	763.0	0.068
*GUSB*	Glucuronidase beta	0.371806	5	272.33	0.070
*PRKCA*	Protein kinase C alpha	−0.33072	5	418.66	0.068

**Table 3:** Biological process analysis of clustered genes associated with milk production.

	Biological Activity Analysis of Clustered Genes
**Cluster 1**	Translation, mitochondrial translational elongation, mitochondrial translational initiation, protein transport.
**Cluster 2**	BMP signalling pathway, regulation of MAPK cascade, fat cell differentiation, carbohydrate metabolic process, cell development, SMAD protein signal transduction, positive regulation of ERK1 and ERK2 cascade, and positive regulation of gene expression.
**Cluster 3**	Regulation of cell cycle, cell proliferation, calcium ion transmembrane transport, peptidyl-serine phosphorylation, and positive regulation of calcium ion transport into cytosol.
**Cluster 4**	Carbohydrate metabolic process, N-acetylglucosamine metabolic process, N-acetylglucosamine catabolic process, N-acetylneuraminate catabolic process, glycogen catabolic process, and lipid biosynthetic process.

**Table 4:** Pathway analysis of clustered genes associated with milk production.

	Pathway Analysis of Clustered Gene
**Cluster 1**	Ribosome, SNARE interactions in vesicular transport.
**Cluster 2**	Rap1 signalling pathway, Ras signalling pathway, Chemokine signalling pathway, PI3K-Akt signalling pathway.
**Cluster 3**	Protein processing in endoplasmic reticulum, MAPK signalling pathway, ErbB signalling pathway, Ras signalling pathway, Rap1 signalling pathway, Calcium signalling pathway, HIF-1 signalling pathway.
**Cluster 4**	Amino sugar and nucleotide sugar metabolism, Glucagon signalling pathway, AMPK signalling pathway.

**Table 5:** miRNAs associated with top genes identified via network analysis using Cluster ONE and CytoNCA.

Target Genes	miRNAs
*RET*	bta-miR-141, bta-miR-181a, bta-miR-194, bta-miR-2410, bta-miR-29b, bta-miR-760-3p, bta-miR-7863
*CXCR4*	bta-miR-2344
*GAPDH*	bta-miR-2340
*CSF1*	bta-miR-10b, bta-miR-204, bta-miR-214, bta-miR-24, bta-miR-375, bta-miR-665, bta-miR-760-3p, bta-miR-7863
*KDR*	bta-miR-375, bta-miR-2320-5p
*VPS54*	bta-miR-146a, bta-miR-22-3p, bta-miR-2413, bta-miR-31
*RAB6B*	bta-miR-149-5p, bta-miR-21-3p, bta-miR-2357, bta-miR-29b, bta-miR-27a-5p, bta-miR-431, bta-miR-491, bta-miR-541, bta-miR-760-3p
*VPS33B*	bta-miR-183, bta-miR-1247-3p, bta-miR-7863
*YKT6*	bta-miR-197, bta-miR-500, bta-miR-133a, bta-miR-133b
*MRPL9*	bta-miR-370, bta-miR-665
*GUF1*	bta-miR-10b, bta-miR-204, bta-miR-214, bta-miR-24, bta-miR-375, bta-miR-665, bta-miR-760-3p, bta-miR-7863

## Data Availability

The datasets used in this study are publicly available and can be accessed from the National Center for Biotechnology Information (NCBI) with BioProject numbers PRJNA148301. Further details on accessing the data can be found on the NCBI website at https://www.ncbi.nlm.nih.gov/bioproject/ (accessed on 9 October 2023).
